# Meso-level ambulatory care and ambulatory care sensitive hospitalizations in Germany: Wiebke Schüttig

**DOI:** 10.1093/eurpub/ckac129.688

**Published:** 2022-10-25

**Authors:** W Schüttig, L Sundmacher

**Affiliations:** Health Economics Department, Technische Universität München, Munich, Germany

## Abstract

Ambulatory care sensitive hospitalizations are widely considered as important measures of access to as well as quality and performance of primary care. In our study, we investigate the impact of spending, process quality and continuity of care in the ambulatory care sector on ambulatory care sensitive hospitalizations in patients with type 2 diabetes. We used observational data from Germany's major association of insurance companies from 2012 to 2014 with 55,924 patients, as well as data from additional regional data sources. We conducted negative binomial regression analyses with random effects at the district level. To control for potential endogeneity of spending and physician density in the ambulatory care sector, we used an instrumental variable approach. In doing so, we adjust for a number of known risk factors for hospitalizations among this patient group. We undertook a Shorrocks-Shapley decomposition to investigate the relative contribution of groups of regressors to the pseudo R2. The results of our analysis suggest that spending in the ambulatory care sector has weak negative effects on ambulatory care sensitive hospitalizations. We also found that continuity of care was negatively associated with hospital admissions. Patients with type 2 diabetes are at increased risk of hospitalization resulting from ambulatory care sensitive conditions. The results of the decomposition analysis for groups of variables indicate that ambulatory care characteristics account for 9.8% of the pseudo R2, morbidity of patients (including gender and age groups) for about 85.5%, and system-related factors of health provision for 4.7%. Our study provides some evidence that meso-level factors such as increased spending and improved continuity of care while controlling for process quality in the ambulatory care sector may be effective ways to reduce the rate of potentially avoidable hospitalizations among patients with type 2 diabetes.

